# OA15.02. Quantitative findings from piloting the LEAP project: an online spirituality based depression intervention for young adults

**DOI:** 10.1186/1472-6882-12-S1-O59

**Published:** 2012-06-12

**Authors:** S Moritz, J Toews, B Rickhi, P Paccagnan, S Malhotra, C Hart, R Maser, J Cohen

**Affiliations:** 1 The Canadian Institute of Natural and Integrative Medicine, Calgary, Alberta, Canada; 2 Department of Psychiatry, University of Calgary, Calgary, Canada

## Purpose

1) To pilot the LEAP Project (http://www.leapproject.com), an online spirituality based depression intervention for young people aged 13-24. 2) To estimate the impact of the intervention on depression severity.

## Methods

This pilot study used a parallel-group randomized controlled assessor-blinded trial design. A total of 46 individuals aged 13-24 with clinically diagnosed unipolar major depression of mild to moderate severity are being recruited in Calgary, Canada and randomized to two study arms: 1. Immediate Intervention Group (eight week online intervention) and 2. Waitlist Control Group (no intervention for 8 weeks followed by the online intervention). Participants were assessed at baseline, 8, 16 and 24 weeks. The main outcome measure (depression severity) was based on the Children Depression Rating Scale (CDRS for participants aged 13 to 18) and the Hamilton Depression Rating Scale (HAM-D for participants aged 19-24).

## Results

Preliminary analysis of 30 participants (20 participants aged 13-18, 10 participants aged 19-24) indicates notable changes in depression severity at 8 weeks in the Immediate Intervention Group compared to the Waitlist Control Group. For those aged 13-18 in the Immediate Intervention Group the CDRS score change at 8 weeks was -13.8 compared to -1.8 in the Waitlist Control Groups (p=0.044). Follow-up scores for this group at 16 and 24 weeks show a further reduction in depression severity compared to the baseline score (CDRS score change at 16 weeks: -21.6 and at 24 weeks: -23.3). For those aged 19-24 in the Immediate Intervention Group the HAM-D score change at 8 weeks was -8.8 compared to -3.6 in the Waitlist Control Groups (p=0.037); 16 and 24 week follow-up scores suggest that the post intervention score was maintained.

## Conclusion

Preliminary results suggest that the LEAP Project can reduce depression severity long term. Data collection is continuing and final results will be available for presentation in May 2012.

